# What do laboratory-forgetting paradigms tell us about use-inspired forgetting?

**DOI:** 10.1186/s41235-021-00300-6

**Published:** 2021-05-07

**Authors:** Paul S. Scotti, Ashleigh M. Maxcey

**Affiliations:** 1 Department of Psychology, The Ohio State University, Columbus, OH USA; 2 Department of Psychology, Vanderbilt University, Wilson Hall, 111 21st Ave S, Nashville, TN 37212 USA

## Abstract

Directed forgetting is a laboratory task in which subjects are told to remember some information and forget other information. In directed forgetting tasks, participants are able to exert intentional control over which information they retain in memory and which information they forget. Forgetting in this task appears to be mediated by intentional control of memory states in which executive control mechanisms suppress unwanted information. Recognition-induced forgetting is another laboratory task in which subjects forget information. Recognizing a target memory induces the forgetting of related items stored in memory. Rather than occurring due to volitional control, recognition-induced forgetting is an incidental by-product of activating items in memory. Here we asked whether intentional directed forgetting or unintentional recognition-induced forgetting is a more robust forgetting effect. While there was a correlation between forgetting effects when the same subjects did both tasks, the magnitude of recognition-induced forgetting was larger than the magnitude of directed forgetting. These results point to practical differences in forgetting outcomes between two commonly used laboratory-forgetting paradigms.

## Introduction

The desire to forget has been motivated clinically (Wilson et al., 2004), and the ability to exert control over memory has been repeatedly demonstrated in the laboratory (Yang et al., 2020). Indeed, establishing reliable, robust laboratory tasks that induce forgetting has been a priority for decades (Roediger & McDermott, 1995). Directed forgetting is one such laboratory procedure that offers the opportunity for subjects to demonstrate that they can remember items that are cued as to-be-remembered and forget items that are flagged as to-be-forgotten (Anderson & Green, 2001; Anderson & Hanslmayr, 2014). Item-method directed forgetting subjects are instructed to remember or forget each item following its presentation (Basden, 1996). List-method directed forgetting instructions to remember or forget follow a list of items (Bäuml et al., 2010; Bjork, 1989). The intentional nature of directed forgetting suggests that it is driven by intentional executive functioning, potentially inhibitory control (Anderson, 2005; Anderson & Hanslmayr, 2014; Basden, 1996; Brandt et al., 2013; Paz-Caballero et al., 2004; Rae et al., 2015; Rizio & Dennis, 2013; Wylie et al., 2008; Yang et al., 2016). Despite the intuitively straightforward appeal of directed forgetting in managing one’s own memory, there are limitations to forgetting tasks that rely on cognitive control. First, directed forgetting cannot overcome memorability (Bainbridge, 2020). That is, 'in spite of one’s efforts, you cannot make yourself remember a forgettable image, or make yourself forget a memorable image' (Bainbridge, 2020, p. 13). Second, when forgetting is measured explicitly, forgetting may not occur using implicit measures (Vuilleumier et al., 2005). Is there a more effective method of forgetting?

In contrast to directed forgetting, recognition-induced forgetting is a laboratory-forgetting task that appears unintentional. Recognition-induced forgetting involves forgetting information in memory as a consequence of retrieving related items (e.g., other items from the same category) (Maxcey & Woodman, 2014) or restudying the same items (Maxcey et al., 2019b). Similar to directed forgetting, recognition-induced forgetting is also a robust phenomenon (Fukuda et al., under review, 2020; Maxcey & Bostic, 2015; Maxcey et al., 2016, 2018; Maxcey et al., 2019a; b; under review; 2020; Megla et al., in press; Rugo et al., 2017; Scotti et al., 2020) with forgetting replicated across a variety of modified paradigms (Bekinschtein et al., 2018; Reppa et al., 2017, 2020; Tan & Jiang, 2019).

As with directed forgetting, a dominant account of recognition-induced forgetting involves inhibitory mechanisms (Anderson, 2003). Despite this similarity with directed forgetting, recognition-induced forgetting has recently been shown to be beyond cognitive control, such that forgetting persists even when participants are given explicit knowledge on how recognition-induced forgetting works and are instructed to prevent this effect before starting the experiment (Maxcey et al., 2019a). The dissociation between directed forgetting and incidental forgetting phenomena (Paller, 1990) like recognition-induced forgetting leaves open the possibility that recognition-induced forgetting may be a more or less robust real-world forgetting phenomenon than directed forgetting.

Use-inspired science is motivated by the potential use or application of the scientific discovery (Stokes, 2011). Is there reason to believe these two forgetting effects have differences that may impact their utility in the laboratory and in real-world applications, such that one produces larger forgetting effects than the other? On the one hand, executive control processes like inhibition have been invoked by theoretical accounts for both directed (Bjork, 1989; Geiselman et al., 1983) and induced forgetting effects (Anderson, 2003; Anderson & Hulbert, 2020; Storm et al., 2007), suggesting that the same underlying mechanism might be involved (but see Sahakyan et al., 2013). This could suggest that no measurable difference in forgetting would be found between directed and recognition-induced forgetting. On the other hand, the opposing directions of processing (intentional vs. incidental) have also been theoretically explained using separate mechanisms of forgetting. For example, directed forgetting may be due to differential processing (e.g., selective rehearsal) of Forget and Remember items (Bjork, 1972; MacLeod, 1999; Wetzel, 1975), whereas recognition-induced forgetting may be due to shifts in familiarity (Raaijmakers, 2016; Raaijmakers & Shiffrin, 1980, 1981). This would suggest that it might be possible to detect a difference in the degree of forgetting produced by these two laboratory-forgetting tasks, leading to different utility outside the laboratory.

What has been done to compare directed forgetting and recognition-induced forgetting? Studies using the directed forgetting paradigm have asked whether memory retrieval operations are the same for intentional and incidental processes (Basden et al., 1993; MacLeod, 1989; Roediger, 1990). However, these studies investigated intentional versus incidental processing by either varying participant instructions or contrasting explicit (e.g., word-stem completion, old/new recognition) and implicit (e.g., word association, speeded word reading, event-related brain potentials) memory measurements. In all cases, participants exerted cognitive control to intentionally forget certain memory items. Existing research has blended aspects of directed and recognition-induced forgetting (Storm et al., 2007), and a review paper has looked at the role of inhibition in three forgetting paradigms, directed forgetting, retrieval-induced forgetting, and the think/no-think paradigm (Bäuml et al., 2010). However, no one has pit directed forgetting and recognition-induced forgetting against each other within the same subjects.

The primary goal of the present study is to directly compare the degree of forgetting caused by item-method directed forgetting and recognition-induced forgetting. Item-method directed forgetting was included because it best allows for the random interleaving of the two forgetting tasks within subjects. Across two experiments we ask, which of these two laboratory-forgetting tasks leads to greater forgetting? A secondary goal is to use individual differences between these two intentional and incidental forgetting effects to demonstrate the degree to which these two constructs are dissociable (Underwood, 1975; Vogel & Awh, 2008). If intentional and incidental operations belong to a single, shared system (Russo & Andrade, 1995), then recognition-induced forgetting and directed forgetting should be correlated and not reliably differ from one another. If intentional and incidental processes are mediated by separate systems (Jacoby, 1984, 1991; Paller, 1990), then recognition-induced forgetting and directed forgetting should not be correlated and the magnitude of forgetting will differ between these two tasks. A third possibility is that there is a shared mechanism during processing that leads to a reliable correlation, but eventually deviates to lead to different magnitudes of forgetting.

Here we take the novel approach of simultaneously probing intentional forgetting (using the directed forgetting procedure where cognitive control is exerted) and incidental forgetting (using the recognition-induced forgetting procedure where cognitive control is not involved) within subjects, allowing us to compare and correlate the resulting magnitudes of forgetting phenomena. Pitting recognition-induced forgetting (no control) against directed forgetting (control) may lead to meaningful use-oriented outcomes, given that goal-directed use of human memory is fundamental to the human experience.

## Experiment 1

### Methods

#### Participants

Experiment 1 included 48 participants (average age 18.88, 30 female, 18 male) who reported normal or corrected-to-normal vision. Participants were undergraduate students at Vanderbilt University who completed the experiment in person in exchange for course credit. Informed consent was obtained through the appropriate Institutional Review Board. No participants were excluded.

We chose this sample size because (1) in the original recognition-induced forgetting paper by Maxcey and Woodman (2014) the smallest effect size measured in Experiment 1 was *d*_*z*_ = 1.376 (*N* = 12 to observe a power of 0.99) and (2) the first demonstration of item-method directed forgetting was conducted by Muther (1965), where effect size was not reported but they reported a *p* value of < 0.001 with 12 participants. Together, these sample sizes are likely too low due to winner’s curse (Button et al., 2013) so we quadrupled the suggested sample size to 48 participants to ensure adequate power.

#### Stimuli & procedure

**Fig. 1:**
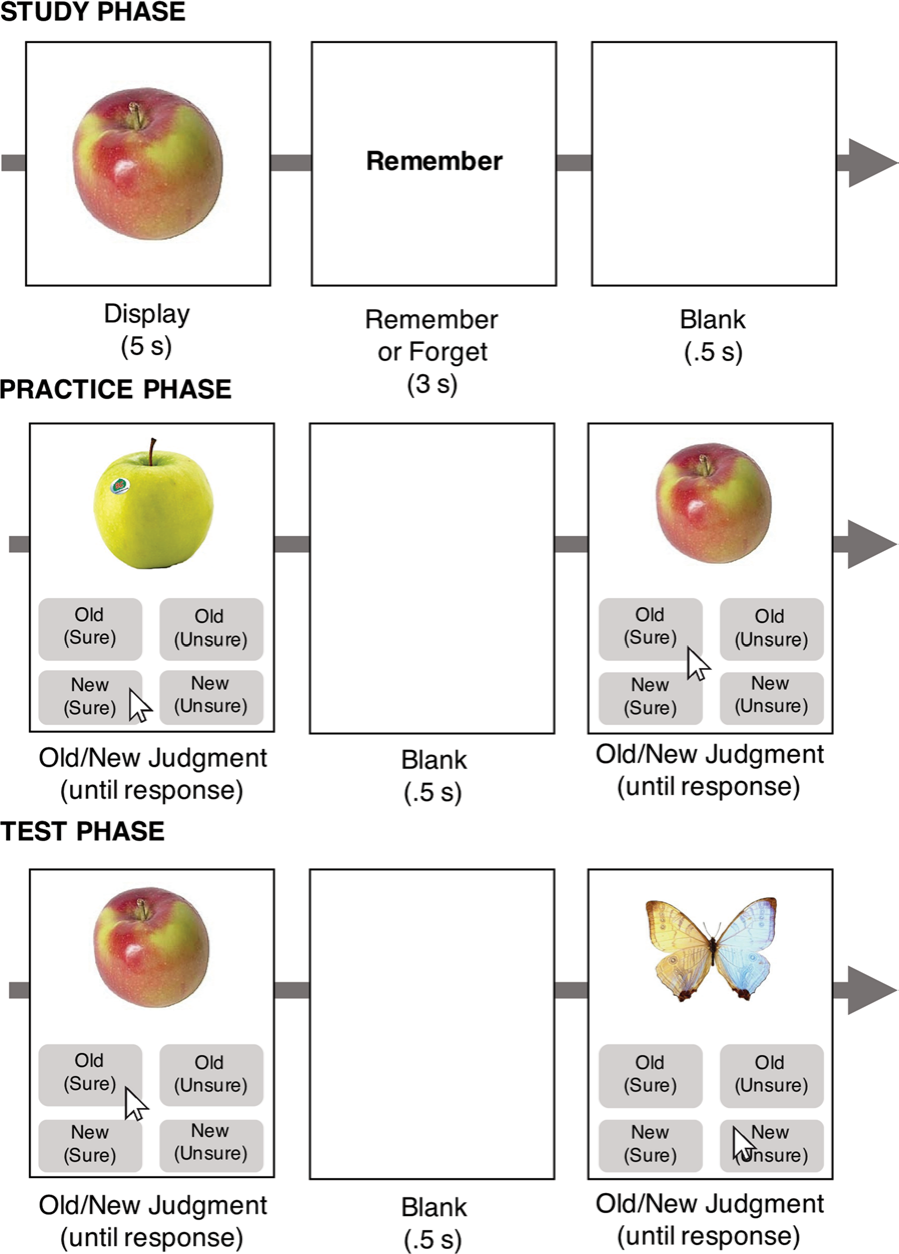
Experiment 1 Methods. The experiment began with the study phase. Participants were presented with 90 objects followed by either a cue to remember (60 trials) or forget (30 trials) the object. Next, in the practice phase, participants were presented with 15 objects from the study phase, on two separate trials (30 total trials), and 30 novel objects drawn from the same categories and asked to make an old-new recognition judgment. Participants had four possible responses, shown here. Finally, in the test phase, the task was identical to the practice phase, but the specific objects differed. All 90 objects from the study phase, along with 90 novel objects were presented

Experiment 1 (Fig. 1) can be viewed online at http://maxceylab.github.io/expts/df/GeneralProcedure_Lab.html.

The total stimulus set from Experiment 1 consisted of 15 object categories, with 18 exemplars in each category (available on OSF https://osf.io/tcfnd/).

##### Study phase

In the study phase, there were 90 trials. The 90 trials consisted of six exemplars from each of the 15 object categories. The 90 trials are comprised of 60 Remember items (ten object categories) and 30 Forget items (five object categories). The unique combination of directed forgetting and recognition-induced forgetting tasks into one experiment necessarily involved twice as many Remember-cued items as Forget-cued items (response bias, if present, can be accounted for using signal detection theory, and if a bias elicited more frequent ignoring of instructions, then the post-experiment survey we implement in Experiment 2 should detect this). Forget items were followed by a cue to forget that item. All other items were followed by a cue to remember. The categories assigned to each cue were randomly determined per subject. Memory for all the items was tested in a surprise memory test at the end of the experiment.

Participants may have noted that the Forget cue followed the same category of items and preemptively not encoded those items. This is unlikely because (1) in E1 we find a magnitude of directed forgetting that is similar to comparable studies employing item-method directed forgetting with picture stimuli (Quinlan et al., 2010) and, as expected due to the picture superiority effect, smaller than words (MacLeod, 1999), and (2) in E2 we find the magnitude of recognition-induced forgetting is *greater* than directed forgetting. If subjects predicted which items would be followed by the Forget cue and selectively decided to not encode them, recognition-induced forgetting would not be larger than directed forgetting.

##### Practice phase

In the practice phase, subjects engaged in an old-new recognition judgment task. Half of the objects from half of the Remember object categories from the Study phase were randomly selected for the practice phase. The 15 Practiced objects (three objects from five categories) were shown twice, on two separate trials, totaling 30 trials. The remaining 30 trials consisted of new objects equally drawn from the same categories. These 30 Practice Lures were each shown separately. All objects were randomly presented.

Subjects responded to each object in both the practice and test phase by clicking on one of four response buttons (old/new divided by sure/unsure, see Fig. 1).

The design of the practice phase created three object types out of the Remember objects. The 15 Remember objects that were included in the practice phase are Practiced objects. The remaining 15 Remember objects from the same categories that were not included in the practice phase (recall that half of the objects from half of the categories were practiced) are Related objects. The Remember objects from entire categories that were not practiced are Baseline objects. Memory for these three objects types, which were all Remember objects, will be tested in the test phase, along with memory for the Forget objects.

##### Test phase

The test phase involved probing memory for all 90 studied objects (15 Practiced, 15 Related, 30 Baseline, and 30 Forget objects) plus 90 Test Lures (Practice Lures were not used in the test phase) from the same object categories. All objects were sequentially presented in random order. The task was the same as the practice phase, responding to each object by clicking on one of four response buttons (old/new divided by sure/unsure, see Fig. 1).

To ensure we were testing long-term memory representations, participants underwent a 2-min filler task before the practice and test phases, a simple color change detection task using colored squares (e.g., Luck & Vogel, 1997).

#### Data analysis

For the old/new recognition judgment measures, we derived the signal detection measure for the area under the curve (AUC), representing memory discrimination de-confounded from potential response bias incorporating false alarm into the sensitivity measure, separately for each subject and each object type (Baseline, Related, Forget, Practiced). Recognition-induced forgetting was calculated as the difference between AUC measures for Baseline and Related items and directed forgetting was calculated as the difference in AUC measures for Baseline and Forget items.

We selected AUC as the primary measurement, rather than the common signal detection measures of *d*′ or *A*′, because AUC considers the relationship between confidence and accuracy. That is, *d*′ and *A*′ approximate the area under the curve with respect to a single point on the receiver operating characteristic (ROC, old/new is a 2-point scale, providing one hit rate and one false alarm rate) despite our data having three ROC points (due to 4-point confidence scale). In addition, AUC provides an index of discriminability, which does not depend on strong, typically untested, assumptions about the distribution of internal states. Rather, AUC is a measure of ordinal separation of the two distributions indexing target responses and lure responses (Weidemann & Kahana, 2016). We used a nonparametric approach to estimate AUC by linearly interpolating between observed ROC points and then calculating area using trapezoidal integration. Here we also report hit rate and d’ as more standard measurements for recognition memory for comparison, which were largely consistent with the AUC results. In the final test phase, we are comparing 30 Forget, 30 Baseline, and 15 Related items using *t* tests. While it is not ideal to have an unbalanced number of items per trial type, it was necessary for our comparison of simultaneous forgetting tasks and it is not a fundamental problem because *t* tests can accommodate unequal samples sizes.

We also conducted a linear correlation between the magnitudes of recognition-induced forgetting and directed forgetting to see if subjects who experienced the strongest recognition-induced forgetting also experienced the strongest directed forgetting.

*t* Tests and correlations are accompanied by JZS Bayes factors, using the default scale *r* value of 0.707 for *t* tests (http://pcl.missouri.edu/bayesfactor). JZS Bayes factor provides directly interpretable odds or probability that data fits under one hypothesis relative to another. For example, JZS_NULL_ = 3.0 means that the null hypothesis is three times as likely as the alternative.

### Results

**Fig. 2:**
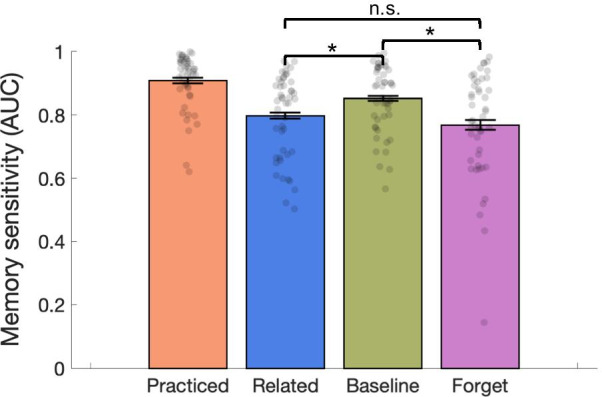
Experiment 1 Results. Directed forgetting (Baseline—Forget) and recognition-induced forgetting (Baseline—Related) were both significant. Error bars are 1/− 1 within-subjects SEM

Figure 2 illustrates the results from Experiment 1 in AUC. Hit rate across object type was Practiced 0.869, Baseline 0.764, Related 0.650, and Forget 0.608. We found reliable directed forgetting, with memory for Forget items worse than memory for Baseline items across AUC (*t*(47) = 4.02, *p* < 0.001, *d* = 0.58, JZS_ALT_ = 118.3), hit rate (*t*(47) = 4.59, *p* < 0.001, *d* = 0.66, JZS_ALT_ = 638.9), and *d′* (*t*(47) = 4.43, *p* < 0.001, *d* = 0.64, JZS_ALT_ = 394.1). We also found reliable recognition-induced forgetting, with worse memory for Related objects relative to Baseline across AUC (*t*(47) = 4.97, *p* < 0.001, *d* = 0.72, JZS_ALT_ = 2,065.3), hit rate (*t*(47) = 5.57, *p* < 0.001, *d* = 0.80, JZS_ALT_ = 13,948.0), and *d′* (*t*(47) = 5.52, *p* < 0.001, *d* = 0.80, JZS_ALT_ = 11,870.4). Directed forgetting magnitude and recognition-induced forgetting magnitudes were not significantly different across subjects using AUC (*t*(47) = 1.28, *p* = 0.208, JZS_NULL_ = 2.271), hit rate (*t*(47) = 1.06, *p* = 0.295, JZS_NULL_ = 2.902), or *d′* (*t*(47) = 1.14, *p* = 0.261, JZS_NULL_ = 2.668). Practiced items were remembered significantly better than all other object types (all *t*s > 5).

Having successfully produced both the expected directed forgetting and recognition-induced forgetting patterns of results, we next turned to the correlation between these forgetting effects. We found that recognition-induced forgetting was not reliably correlated with directed forgetting across AUC (*r* = 0.041, *p* = 0.783, JZS_ALT_ = 1.03), hit rate (*r* = 0.015, *p* = 0.921, JZS_ALT_ = 1.00), or *d′* (*r* = 0.161, *p* = 0.275, JZS_ALT_ = 1.73).

### Discussion

In Experiment 1, we found no difference in the magnitude of recognition-induced forgetting and directed forgetting and no correlation between the two forgetting effects. The wide variability in subjects’ response to items that were cued to forget (e.g., a few participants had a hit rate of 0 for Forget items) suggests variability in subjects’ interpretation of the experiment instructions. For example, it may be that a subset of subjects was intentionally selecting New even though they recognized the item, because they knew it was associated with the ‘Forget’ cue.

## Experiment 2

**Fig. 3:**
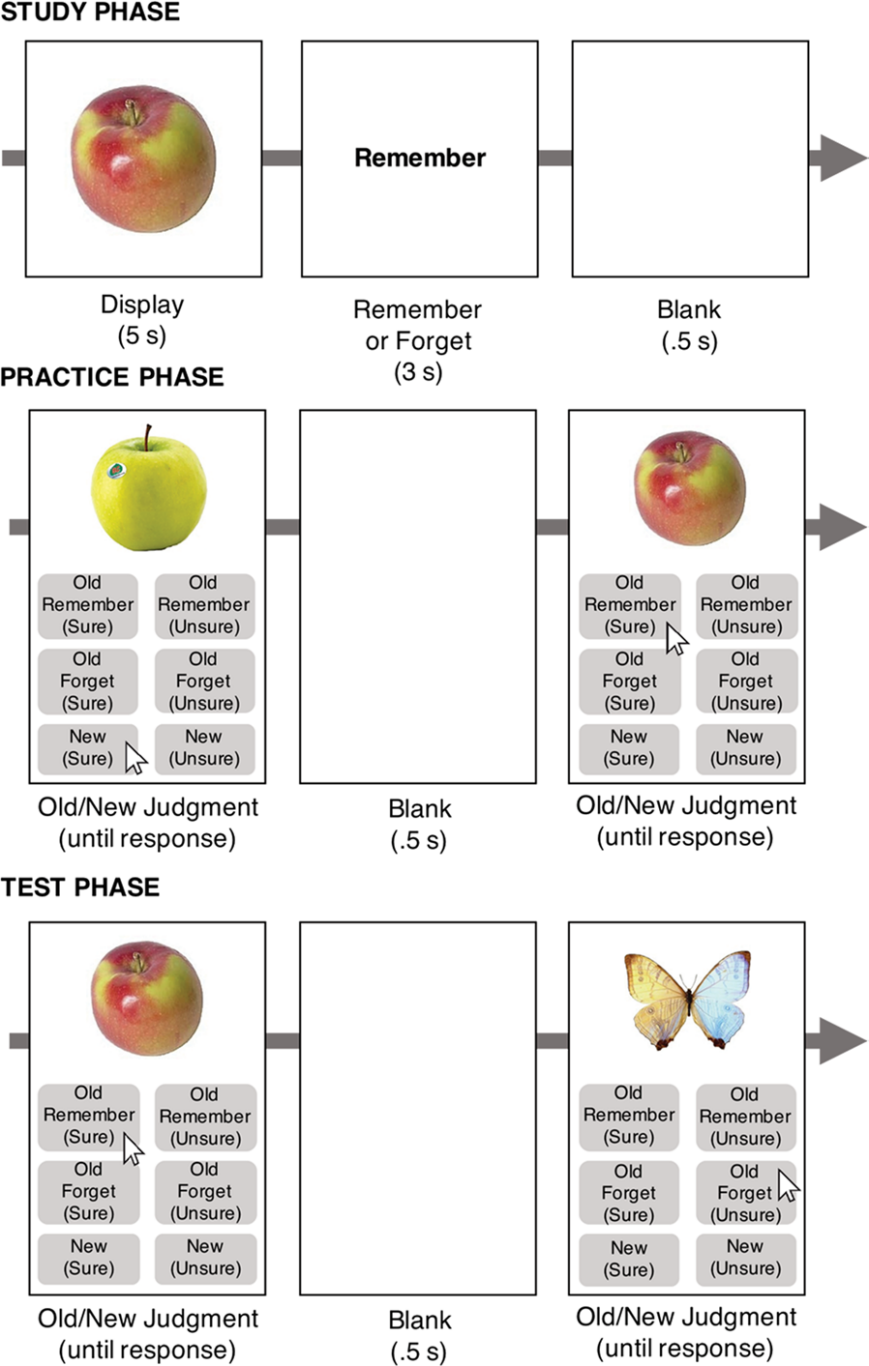
Experiment 2 Methods. The stimuli, task, and three phases were identical to Experiment 1 with the following exception. Participants had six response choices instead of four, pictured above. Not pictured: in a post-experiment survey participants were asked whether they believed the Forget items would be tested

The purpose of Experiment 2 was to determine whether evidence of equivalent forgetting across forgetting effects and of no relationship between directed forgetting and recognition-induced forgetting from Experiment 1 would replicate and extend to circumstances under which we could more confidently ensure subjects were following the cue to Forget and responding appropriately based on their available memory representations. To correct for this potential demand characteristic or general confusion in how to respond to items that are remembered but were cued to forget, we implemented two changes in Experiment 2. We offered subjects additional available responses (akin to a 'tagging' procedure, e.g., MacLeod, 1999; Thompson et al., 2011) shown in Fig. 3 and introduced a post-experiment survey that probed participants reported compliance with the remember and forget cues (in a similar vein as Foster & Sahakyan, 2011).

## Methods

Experiment 2 was identical to Experiment 1 with the following exceptions.

### Participants

Subjects originally consisted of 96 new participants (average age 18.906, 29 male, 67 female). Participants were undergraduate students at Vanderbilt University who completed the experiment in exchange for course credit. Based on their post-experiment report that they expected to be tested on the Forget items before starting the experiment (rationale explained in post-experiment survey section below), 44 participants were excluded, leaving 52 participants (average age 18.846, 16 male, 36 female) for subsequent analyses. We ran a post hoc power analysis to ensure our correlation was sufficiently powered, especially considering the large number of excluded subjects. For a two-tailed correlation between directed forgetting and recognition-induced forgetting using an assumed *ρ* = 0.3 (not using the data from the initial sample of subjects, which would be circular), power of 0.8, and an alpha of 0.05 (Faul et al., 2007), the power analysis suggested a sample size of 84 subjects. Therefore, we continued collecting data until we had 84 subjects who reported believing that Forget cued items would not be tested in the post-experiment survey. Due to COVID-19, this involved 64 additional subjects collected online via Amazon Mechanical Turk (average age 31.38, 41 male, 23 female) in exchange for monetary compensation. The collapsed dataset is reported below. The two samples are analyzed separately on Open Science Framework (https://osf.io/tcfnd/).

### Procedure

Experiment 2 was run online and can be viewed online at https://maxceylab.github.io/expts/df/GeneralProcedure_Lab2.html. In Experiment 2 (Fig. 3), subjects responded to objects with six response buttons rather than four, such that old responses were further divided by whether it was followed by a Forget/Remember instruction. These additional buttons helped ensure that participants did not falsely believe that they should respond New to an object that they recalled was followed by the instruction to forget.

At the end of the experiment, subjects were asked whether they believed that Forget-cued items would be subsequently tested. Specifically, they were asked, 'When you first studied the pictures, did you expect that you might be tested on pictures that were followed by the instruction to Forget?'.

## Results

### Post-experiment survey

Although it is not standard practice in directed forgetting experiments to ask subjects whether they believed that Forget items would be tested (but see Foster & Sahakyan, 2011), variability in responses from Experiment 1 led us to believe there was response confusion (e.g., how should a subject respond to an object that they remembered being cued to Forget?) or disregard of the Forget instruction because of an assumption that Forget items would be tested. We implemented the post-experiment survey to detect subjects who fell into the latter category. We found that nearly half of the participants may have disregarded the Forget cue, as 76/160 (47.5%) subjects reported believing that the Forget items would be tested. This high rate of distrust in the cue directing subjects to forget suggests that the assumption typically made in directed forgetting procedures that subjects will follow the cue may not be justified.

Based on their post-experiment report that they expected to be tested on the Forget items, 76 participants were excluded from the primary analyses reported below, leaving 84 participants (average age 23.58, 36 male, 48 female).

Memory for the Forget items was statistically indistinguishable between the subjects who believed Forget items would not be tested (0.76) and subjects who believed Forget items would be tested (0.75, *t*(158) = 0.369, *p* = 0.712, JZS_NULL_ = 5.50). This may mean that forgetting in directed forgetting is not driven by belief in the cue, and thus not driven by an intentional response. Alternatively, it may be that despite thinking they may be tested on Forget items, subjects still enacted an intentional forgetting strategy (Foster & Sahakyan, 2011). Regardless of what these subjects were doing in response to the Forget cue, the purpose of Experiment 2 was to replicate and extend Experiment 1 while ensuring that the results of Experiment 1 could not be explained by demand characteristics or by participant confusion over how to classify a known Forget item. This ‘purer’ measure of directed forgetting was achieved by introducing additional response buttons to reduce or eliminate response confusion and using the post-experiment survey to identify and exclude participants who told us at the end of the experiment that they believed Forget items would be tested. We therefore initially report below the same analyses as Experiment 1 when only including participants who responded that they believed Forget items would not be tested, followed by an analysis of the full dataset. We have made available the full dataset for both experiments on the Open Science Framework (https://osf.io/tcfnd/).

While some may argue that excluding subjects based on a post-experiment survey is inappropriate (Foster & Sahakyan, 2011), the admission of subjects that they did not follow the direction to forget seems like a fundamental exclusion criterion when comparing intentional and incidental forgetting. Indeed, according to Sahakyan and Foster (2016, p. 3) themselves, paradigms used to study intentional forgetting share the trait that 'people are instructed to exert control over the contents of their mind by engaging in behaviors or processes that limit the accessibility to unwanted information.' Thus analyzing data from subjects who likely did not engage in such behaviors is a conservative approach to ensuring that our measure of directed forgetting is truly reflecting an intentional process.

### Directed forgetting and recognition-induced forgetting in subjects who did believe the Forget cue (*N* = 84, exclusions based on post-experiment survey)

**Fig. 4:**
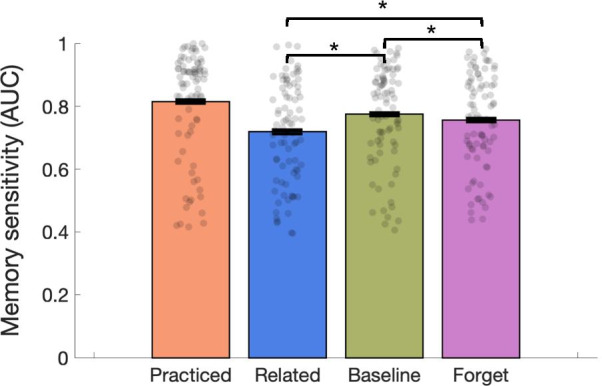
Experiment 2 Results, showing only the subjects who reported they did not believe that Forget items would ever be tested (N = 84). Replicating Experiment 1, directed forgetting (Baseline—Forget) and recognition-induced forgetting (Baseline—Related) were both significant. Unlike Experiment 1, recognition-induced forgetting was larger than directed forgetting

Old (Forget) and Old (Remember) responses were collapsed across respective confidence levels (unsure or sure) because the intention behind the additional buttons was to ensure participants were following instructions rather than test for differences in these responses (see Table 1 for trial distribution). Hit rates by object type were Practiced 0.860, Baseline 0.791, Related 0.690, and Forget 0.769. Replicating Experiment 1, we found reliable directed forgetting (Fig. 4), with memory for Forget items lower than Baseline across AUC (*t*(83) = 2.41, *p* = 0.018, *d* = 0.26, JZS_ALT_ = 1.83), but not using hit rate (*t*(83) = 1.42, *p* = 0.158, JZS_NULL_ = 3.16) or *d′* (*t*(83) = 1.05, *p* = 0.298, JZS_NULL_ = 4.89). We found reliable recognition-induced forgetting, with memory for Related objects lower than memory for Baseline objects across AUC (*t*(83) = 5.37, *p* < 0.001, *d* = 0.59, JZS_ALT_ = 20,213.5), hit rate (*t*(83) = 5.35, *p* < 0.001, *d* = 0.58, JZS_ALT_ = 18,703.3) and *d′* (*t*(83) = 4.43, *p* < 0.001, *d* = 0.48, JZS_ALT_ = 625.2). Practiced items were again remembered significantly better than all other object types (all *t*s > 4).

**Table 1:** Proportion of trials in which participants pressed each of the four possible responses in the test phase of Experiment 1 (Old Sure, Old Unsure, New Sure, New Unsure) and each of the six possible responses in Experiment 2 (Old Remember Sure, Old Remember Unsure, Old Forget Sure, Old Forget Unsure, New Sure, New Unsure). Experiment 2 is separated by the 84 participants who are included in the primary analyses (denoted Experiment 2 (Included)) and the 76 participants who were excluded from the primary analyses based on post-experimental survey responses (denoted Experiment 2 (Excluded))

	Old sure	Old unsure	New sure	New unsure		
Experiment 1	0.316	0.102	0.392	0.190		

	Old remember sure	Old remember unsure	Old forget sure	Old forget unsure	New sure	New unsure
Experiment 2 (included)	0.243	0.082	0.120	0.102	0.273	0.181
Experiment 2 (excluded)	0.269	0.082	0.141	0.081	0.289	0.137

Results differed from Experiment 1 in that in Experiment 2 we observed significantly larger recognition-induced forgetting than directed forgetting across AUC (*t*(83) = 3.45, *p* < 0.001, *d* = 0.38, JZS_ALT_ = 26.6), hit rate (*t*(83) = 4.03, *p* < 0.001, *d* = 0.44, JZS_ALT_ = 161.4), and *d′* (*t*(83) = 3.24, *p* = 0.002, *d* = 0.35, JZS_ALT_ = 14.6). In the 84 subjects who believed Forget items would not be tested, recognition-induced forgetting was reliably correlated with directed forgetting across AUC (*r* = 0.315, *p* = 0.004, JZS_ALT_ = 57.30), hit rate (*r* = 0.328, *p* = 0.002, JZS_ALT_ = 84.99), and *d′* (*r* = 0.313, *p* = 0.004, JZS_ALT_ = 54.86).

### Recognition-induced forgetting and directed forgetting across all subjects (*N* = 160, no exclusions based on post-experiment survey)

Across all 160 participants, hit rates by object type were Practiced 0.858, Baseline 0.800, Related 0.720, and Forget 0.774. We found reliable directed forgetting with memory for Forget items lower than Baseline across AUC (*t*(159) = 2.67, *p* = 0.008, *d* = 0.21, JZS_ALT_ = 2.71), hit rate (*t*(159) = 2.46, *p* = 0.015, *d* = 0.19, JZS_ALT_ = 1.63) and *d′* (*t*(159) = 2.46, *p* = 0.015, *d* = 0.19, JZS_ALT_ = 1.63). We also found reliable recognition-induced forgetting, with memory for related objects lower than memory for Baseline objects, across AUC (*t*(159) = 6.54, *p* < 0.001, *d* = 0.52, JZS_ALT_ = 11,025,967), hit rate (*t*(159) = 6.60, *p* < 0.001, *d* = 0.52, JZS_ALT_ = 14,968,651), and *d′* (*t*(159) = 5.89, *p* < 0.001, *d* = 0.47, JZS_ALT_ = 448,231). The magnitude of recognition-induced forgetting was significantly larger than directed forgetting for AUC (*t*(159) = 2.67, *p* = 0.008, *d* = 0.21, JZS_ALT_ = 2.71), hit rate (*t*(159) = 4.34, *p* < 0.001, *d* = 0.34, JZS_ALT_ = 558.3), and *d′* (*t*(159) = 3.71, *p* < 0.001, *d* = 0.29, JZS_ALT_ = 58.0). Recognition-induced forgetting was reliably correlated with directed forgetting across AUC (*r* = 0.358, *p* < 0.001, JZS_ALT_ = 37,112), hit rate (*r* = 0.386, *p* < 0.001, JZS_ALT_ = 245,323), and *d′* (*r* = 0.438, *p* < 0.001, JZS_ALT_ = 13,761,770).

## Discussion

In Experiment 2, we added response buttons to help ensure that participants understood directed forgetting instructions and we introduced a post-experiment survey to determine which subjects may not have followed our Forget instruction to allow us the ‘purest’ measure of directed forgetting. By isolating just those subjects who believed Forget items would not be tested, we observed that the magnitude of recognition-induced forgetting was larger than the magnitude of directed forgetting across participants. This replicated when including all participants in the analysis. Recognition-induced forgetting was also correlated with directed forgetting, suggesting the two may be tapping into a shared underlying memory signal.

## General discussion

In the present use-inspired study, we asked whether one of two laboratory-forgetting tasks, directed forgetting and recognition-induced forgetting, leads to larger forgetting effects. The experimental design allowed for a direct comparison between the magnitudes of recognition-induced forgetting and directed forgetting. In Experiment 1, we implemented a within-subjects design that combined an item-based directed forgetting task with the recognition-induced forgetting task in the same paradigm. We did not find any difference between the magnitudes of recognition-induced forgetting and directed forgetting. However, that result may have been influenced by variability introduced by response confusion, disregard of the instruction to forget, or insufficient statistical power due to a small sample size.

In Experiment 2, we found that recognition-induced forgetting is a larger forgetting effect than directed forgetting. Further, in Experiment 2, only approximately half the subjects reported believing Forget items would not be tested. We observed weaker directed forgetting effects among Experiment 2 participants who reported believing the Forget items would not be tested (mean AUC of included Baseline—Forget = 0.0186) compared to the participants in Experiment 1 (Baseline—Forget = 0.0839, independent samples *t* test, *t*(130) = 3.48, *p* < 0.001, *d* = 0.63, JZS_ALT_ = 39.8). The observation that a few participants in Experiment 1, but no participants in Experiment 2, had a hit rate of 0 for Forget items (despite more than tripling the sample size in Experiment 2), along with the reliable decrease in directed forgetting when additional response buttons were introduced in Experiment 2 suggests that directed forgetting is susceptible to inflated effect size from demand characteristics and/or response confusion.

One may argue that the use of semantically related objects in this design could trump the instruction to forget, leading to smaller directed forgetting effects, as has been shown with related word stimuli (Golding et al., 1994). However, if the use of semantically related stimuli here did weaken directed forgetting, that only further supports the point that recognition-induced forgetting is a more robust method for inducing forgetting because pre-existing semantic relationships do not weaken recognition-induced forgetting and such semantic relationships happen frequently in the real world (e.g., pumpkins in a pumpkin patch, kids on a playground, chairs in a lecture hall). Taken together, concerns regarding demand characteristics (but see MacLeod, 1999), response confusion, and the larger magnitude of recognition-induced forgetting suggest that a paradigm that implicitly induces forgetting, such as the recognition-induced forgetting paradigm (Maxcey & Woodman, 2014) employed here, may be the preferred method for use-inspired studies that are more interested in eliciting large magnitudes of forgetting rather than how such forgetting is elicited (intentionally or incidentally).

The intentional nature of directed forgetting and the incidental nature of recognition-induced forgetting appear to illustrate two distinct forgetting effects (e.g., inhibition and selective rehearsal). The secondary question we asked here was whether the same underlying mechanism mediates these forgetting tasks. If recognition-induced forgetting and directed forgetting are driven by the same underlying mechanism, then they should be experimentally correlated such that subjects with large recognition-induced forgetting effects also demonstrate large directed forgetting effects. On the other hand, if recognition-induced forgetting and directed forgetting are not mediated by the same underlying mechanism, then they should be experimentally uncorrelated. In Experiment 1, we found no reliable correlation between directed forgetting and recognition-induced forgetting. However, in Experiment 2, when sample size was increased and response confusion was reduced, we found a reliable correlation between these two forgetting effects. Future work will need to be done to confirm a potential shared mechanism behind the two forgetting effects as we did not include a baseline comparison condition of the same participants engaging in two directed forgetting tasks or two recognition-induced forgetting tasks. This condition is critical to calculating a baseline correlation of the same subject participating in the same task across the two sessions. Another important future direction is why recognition-induced forgetting appears more robust than directed forgetting despite potentially sharing an underlying forgetting mechanism. Finally, here we argue that when forgetting is the goal, induced forgetting may be the preferred method. However, this is not to undermine the importance of studying directed forgetting due to the obvious intentional deployment of cognitive control over everyday remembering and forgetting.

## Data Availability

Experiment 1 can be viewed online at http://maxceylab.github.io/expts/df/GeneralProcedure_Lab.html. Experiment 2 can be viewed online at https://maxceylab.github.io/expts/df/GeneralProcedure_Lab2.html. The full dataset and stimulus set for both experiments can be found on the Open Science Framework (https://osf.io/tcfnd/).
